# Eosinophilic myocarditis: two case reports and review of the literature

**DOI:** 10.1186/1756-0500-6-538

**Published:** 2013-12-17

**Authors:** Jacques Rizkallah, Angela Desautels, Amrit Malik, Shelley Zieroth, Davinder Jassal, Farrukh Hussain, Francisco Cordova

**Affiliations:** 1 Section of Cardiology, Department of Medicine, St Boniface Hospital and University of Manitoba, Y3005-409 tahé avenue, R2H 2A6, Winnipeg, Manitoba, Canada

**Keywords:** Eosinophilia, Myocarditis, Endomyocardial, Biopsy

## Abstract

**Background:**

Eosinophilic myocarditis is a rare and often under-diagnosed subtype of myocarditis with only around 30 cases published in the medical literature. In this article we present two patients with eosinophilic myocarditis with the aim to demonstrate the often elusive nature of the disease and present the current scientific literature on this topic.

**Case presentation:**

A 76 years old Caucasian gentleman and a 36 years old Aboriginal gentleman both presenting with heart failure symptoms were eventually diagnosed with eosinophilic myocarditis after extensive evaluation. Their presentation, assessment, and medical management is explored in this article.

**Conclusions:**

Eosinophilic myocarditis remains a rare and likely under-diagnosed subtype of myocarditis. The key features of this disease include myocardial injury in the setting of non-contributory coronary artery disease. Endomyocardial biopsy remains the definitive gold standard for diagnosis of noninfectious eosinophilic myocarditis. Non-invasive cardiac imaging in the setting of peripheral eosinophilia can be strongly suggestive of eosinophilic myocarditis with potential for earlier diagnosis. Failure to diagnose eosinophilic myocarditis and the delay of therapy may lead to irreversible myocardial injury. Therapies for this disease have yet to be validated in large prospective studies.

## Background

Myocarditis refers to heart muscle inflammation secondary to direct external antigen exposure such as viruses, bacteria, parasites, and drugs or to autoimmune activation against self-antigens. Traditionally the diagnosis of myocarditis was based on the histological Dallas criteria on endomyocardial biopsy which mandates the visualization of inflammatory cells and myocardial necrosis on the same microscopic section; if concomitant necrosis is not detected the diagnosis of myocarditis is considered borderline. Given the limitations of endomyocardial biopsies, in particular its low sensitivity from the often patchy nature of the disease and potential procedural risks, more recent and broader definitions of myocarditis were introduced. These encompass a hybrid of clinical, laboratory, and imaging criteria that may help secure the diagnosis and forgo the need for a biopsy in all cases [1].

The prevalence of myocarditis in general is not well established given the lack of consensus on its diagnostic criteria in the scientific community. As such, among unselected autopsy series, its prevalence is as high as 1 to 5% [1]. The most common causes of myocarditis include infectious and autoimmune etiologies [1]. Eosinophilic myocarditis (EM) is a rare subtype of myocarditis characterized by focal or diffuse myocardial inflammation with infiltrating eosinophils and is often associated with peripheral blood eosinophilia [2,3]. To date there are less than 30 published case reports of EM and include patients ranging from 2 to 83 years of age [2]. Given the rarity of this form of myocarditis, it is often under-recognized and first discovered on postmortem examination [4]. EM was observed in 0.5% of unselected autopsy series and in more than 20% of explanted hearts from heart transplant recipients secondary to drug-induced hypersensitivity [4].

In this review article we present two cases of eosinophilic myocarditis and outline the current scientific literature on this topic including its pathophysiology, diagnosis, and recommended therapy.

## Case presentation

### Case 1

**Figure 1:**
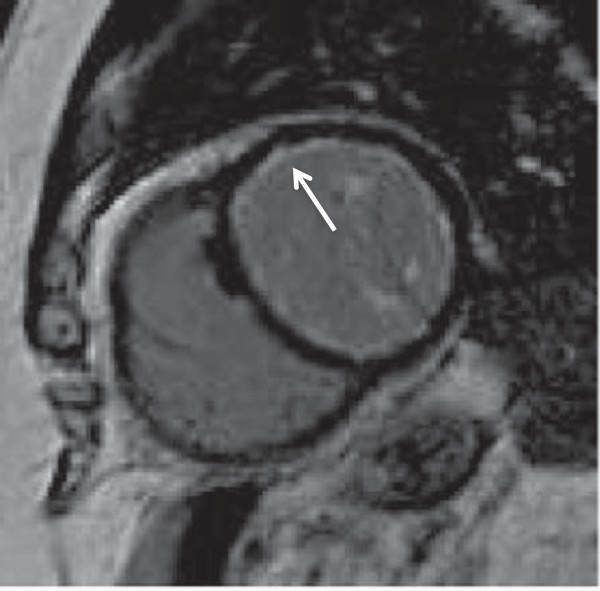
Magnetic resonance imaging with arrow depicting circumferential subendocardial delayed enhancement on the short axis view.

**Figure 2:**
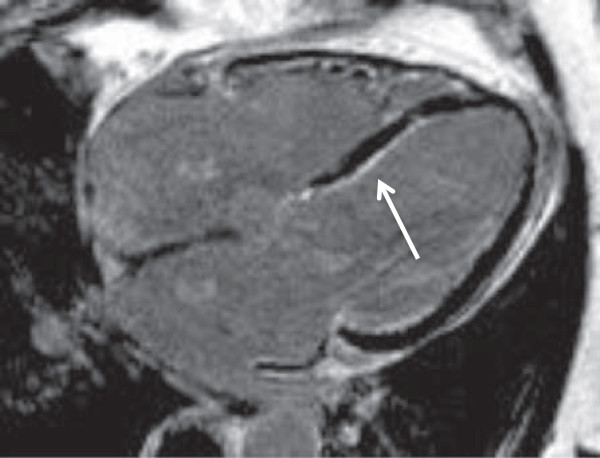
Magnetic resonance imaging with arrow depicting subendocardial delayed enhancement in the 4 chamber axial view.

A 76-year-old Caucasian gentleman with a history of hypertension and asthma presents with history of sharp and pleuritic chest pain radiating to the shoulders with associated dyspnea with gradual deterioration of functional status to New York heart Association (NYHA) functional class III. Laboratory investigations revealed a normal creatine kinase (CK), elevated troponin T at 2.74 ug/L, C– reactive protein (CRP) at 140 mg/L (normal: 0–8), and eosinophilia at 3.92×105/L. The initial presumptive diagnosis was acute coronary syndrome however coronary angiography revealed no significant coronary artery disease. The patient was found to have global reduction in left ventricular systolic function, ejection fraction (EF) 30-35%, along with mildly impaired right ventricular dysfunction on echocardiography. In addition, an uninfused computed tomography (CT) scan of the chest revealed a small right pleural effusion and multiple small centrilobar nodules in the lungs bilaterally with upper lung zone predominance and ground glass attenuation consistent with pulmonary eosinophilia. Magnetic resonance imaging (MRI) identified mild subendocardial delayed enhancement with a near circumferential distribution raising the possibility of eosinophilic myocarditis (Figures 1, 2). There were no suggestions of underlying infection and stool for ova and parasites as well as serology for trypanosome and strongyloides were negative. A vasculitis screen including anti-neutrophil cytoplasmic antibodies (ANCAs) was negative. There were no recently started medications to suggest a drug induced hypersensitivity reactions and physical exam and history were negative for malignancy.

Given this presentation, a diagnosis of clinically suspected eosinophilc myocarditis, idiopathic hypereosinophilic syndrome subtype (HES) in particular, with possible pulmonary involvement was made. The patient declined to undergo a confirmatory endomyocardial biopsy. In addition to the standard medical management of heart failure he was started on oral prednisone at 60 mg per day with 10 mg tapering doses per week to a baseline maintenance dose of 10 mg per day. Unfortunately he showed little to no recovery in cardiac function and is currently being treated medically for his heart failure and followed as an out-patient in the heart failure clinic.

### Case 2

**Figure 3:**
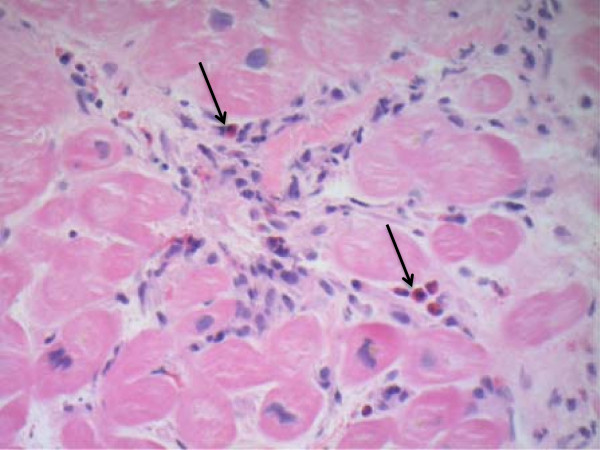
Endomyocardial biopsy of a patient with eosinophilic myocarditis (infiltrating eosinophils depicted by arrows).

A 36-year-old male presents with a two-month history of shortness of breath on exertion progressing to NYHA class III symptoms. His history and physical examination were otherwise unremarkable aside from this new onset heart failure. Laboratory investigations revealed mildly elevated white blood cell (WBC) count with mild elevation in the absolute eosinophil count (1.7×10^5^/L; normal range 0.0-0.4×10^5^/L). Echocardiography demonstrated severe biventricular dysfunction with left ventricular EF <20% and subendocardial delayed enhancement was identified on cardiac MRI consistent with myocarditis. Coronary angiography was unremarkable and endomyocardial biopsy demonstrated active myocarditis with intact and degranulating interstitial eosinophils; greater then 5–8 cells per biopsy specimen (Figure 3). Since there was no evidence of infection, vasculitis, malignancy, or drug induced hypersensitivity reactions the diagnosis of idiopathic eosinophilic myocarditis was established. In addition to standard heart failure treatment, 1 g/day of IV methylprednisolone was initiated for three days followed by two weeks of oral prednisone at 50 mg/day with slow taper. The maintenance dose of steroids was unfortunately discontinued prematurely secondary to corticosteroid-induced psychosis and the development of a concurrent cellulitis and suspicion of pneumonia precluded alternative immunosuppressive treatment. Only mild improvement in cardiac function was observed; the patient is currently being treated medically for his heart failure and followed as an out-patient at the heart failure clinic.

## Discussion

When the diagnosis of myocarditis is strongly suspected confirming the etiology as eosinophilic in nature may be challenging short of endomyocardial biopsy given the rarity of the disease and its vague clinical presentation. Patients with EM may indeed present with various signs and symptoms including fever, chills, malaise, weight loss, acute coronary syndrome-like features, heart failure, tachy- or brady-type arrhythmias, and sudden death [4-6]. To date there are no universally accepted guidelines for the diagnosis of EM but as the disease becomes better recognized so will its characterization. The Japanese Circulation Society Task Force Committee on Acute and Chronic Myocarditis published helpful guidelines for the diagnosis and treatment of EM [7]; essential diagnostic features include eosinophilia > 500/μL, cardiac symptoms, elevated cardiac enzymes, electrocardiogram (ECG) changes, and cardiac dysfunction on ultrasonography, especially in the setting of unremarkable coronary angiography. Definitive diagnosis requires an endomyocardial biopsy [8].

In EM, the eosinophils may be associated with myocardial inflammation in three distinct forms [1]. The first is a hypersensitivity reaction to a foreign antigen known as allergic esosinophilic myocarditis and is often drug induced [1]. Myocardial inflammation may also be triggered in association with systemic eosinophilc disorders such as Churg-Strauss. Lastly, EM may present in the form of fulminant necrotic myocarditis of unclear etiology [1]. Clinical disorders that may result in both hypereosinophilia and endomyocardial injury include idiopathic hypereosinophilic syndrome, malignancies, parasitic infections, vasculitic and granulomatous diseases, tropical endomyocardial diseases, drug reactions, and transplant rejections [9]. A paramount diagnostic feature of EM on endomyocardial biopsy includes the detection of myocardial eosinophils; these inflammatory cells are rarely detected in normal myocardial interstitium thus their presence is considered pathologic for EM [10]. Regardless of the underlying etiology of EM, whether related to the HES or a parasitic infection, eosinophil-mediated cardiac injury occurs in a similar fashion and ranges from early necrosis to subsequent thrombosis and fibrosis [11]. On a macroscopic level this type of myocardial injury can translate into variable degrees of focal or diffuse structural abnormalities including systolic or diastolic dysfunction, mural thrombi, micro-abscesses, ventricular wall aneurysms, and rupture [10,12,13]. The sensitivity of endomyocardial biopsy based on autopsy specimens was estimated to be around 54% given the often patchy nature of the disease and this is likely even lower in the beating heart due to the technical difficulties of the biopsy procedure [14]. A minimum of 5 right ventricular samples should be obtained, however this is based on transplant rejection data for which endomyocardial biopsy is very sensitive and is not standardized for the detection of myocarditis [15]. Additionally, studies have shown significant interobserver variability in the interpretation of endomyocardial biopsy specimens in diagnosis of myocarditis [16]. Although life-threatening complications such as right ventricular perforation are reported to be less than 1%, the overall complication rate including tricuspid regurgitation, occult pulmonary embolism, transient arrhythmias and access site hematoma is as high as 6% [15].

The presence of peripheral eosinophilia may heighten the suspicion for EM but relying solely on this laboratory parameter to diagnose EM and judge treatment response may be misleading. In essence, some patients with sustained eosinophilia never develop cardiac disease and others with biopsy confirmed EM never develop eosinophilia throughout the course of their disease [4,11]. The detection of eosinophil cationic protein (ECP), one of several toxic proteins derived from the degranulation of eosinophils, may be an adjunct to the diagnostic potential of peripheral eosinophilia [17]. Arima and colleagues were the first to report the utility of serum levels of ECP in the diagnosis and treatment response of five patients with EM showing normalization of ECP levels with disease regression [17]. These observations suggest that ECP may in part be responsible for the pathophysiology of EM and that the number of myocardial degranulated eosinophils rather than the total number of eosinophils may be of greater diagnostic significance [17].

Non-invasive cardiac imaging in myocarditis, such as echocardiography, nuclear imaging with gallium^67^- or indium^111^-labeled antimyosin antibodies, and MRI can be useful in assessing myocardial dysfunction although there are no specific features that help establish the diagnosis of EM with certainty as is the case with endomyocardial biopsy [18]. Echocardiography is usually the most readily available imaging modality in most institutions. Common findings on 2D echocardiography include left ventricular dysfunction in up to 69% of cases as evidenced by segmental wall motion abnormalities. Reversible left ventricular hypertrophy can also be observed in 15% of cases while left ventricular cavity dilatation is usually minimal or absent. In addition, only 23% will have right ventricular involvement [18].

Nuclear imaging can be highly sensitive in detecting evidence of myocarditis, such as myocyte necrosis (sen 83%), and although its specificity is only moderate at an estimated 53% it has a high negative predictive value of 92% [18]. Limitations of nuclear imaging include limited tracer availability, poor spatial resolution, and radiation exposure to the patients and staff [19].

Cardiac MRI is a good non-invasive diagnostic imaging alternative, in particular when it comes to its safety, reproducibility, and ability to accurately evaluate cardiac anatomy and function [19]. MRI can nicely depict the common abnormalities noted in myocarditis which include ventricular dysfunction, transient increase in wall thickness and chamber dimensions, pericardial effusion, and inflammatory tissue changes such as edema, capillary leakage, hyperemia, cellular necrosis, and fibrosis [19]. Myocardial edema can be detected using T2-weighted imaging while delayed enhancement imaging allows for the visualization of myocardial fibrosis and inflammation. Esosinophilic myocarditis is typically characterized by extensive myocardial hyperintensity on T-2 weighted imaging along with subendocardial delayed enhancement; mesocardial and epicardial delayed enhancement have also been reported and the extend of delayed enhancement is inversely proportional to LV EF [20]. Subendocardial enhancement can also be identified in the setting of myocardial infarction which should be excluded in the evaluation of a suspect case of myocarditis. The good diagnostic accuracy of MRI in myocarditis was highlighted in pooled controlled trials with a sensitivity and specificity of 67 and 91% respectively along with positive and negative predictive values of 91 and 69% respectively [19].

The initial treatment goal in patients with EM is to ensure hemodynamic stability. Depending on the severity of heart failure and extent of multi-organ involvement patients may require anywhere from intermittent diuresis and analgesia to full cardio-pulmonary support. In addition, reversible and readily treatable etiologies, such as therapy of an underlying parasitic infection or discontinuation of an offending drug, should be identified and addressed as soon as possible. Given the underlying inflammatory nature of EM, therapy with corticosteroids has been successfully documented in various case reports [2-5,9]; it is important to rule out active infection on endomyocardial biopsy prior to initiation of immunosuppression, using viral PCR for instance, to avoid worsening burden of disease [8]. A recent retrospective case series by Kawano and colleagues 20112 was the first to propose initiation and maintenance doses of prednisolone based on disease severity; initial 1 g methylprednisolone pulse dose was reserved for patients with pre-cardiac tamponade, cardiogenic shock, and pulmonary edema as compared to 1 mg/kg/day of prednisolone for more stable patients; a 5-10 mg/day dose of prednisolone was subsequently given to prevent relapse. Not all patients with EM require corticosteroid therapy, especially if the disease severity is very mild [2,21]. There remains little consensus in the use, dose, or duration of corticosteroids in the setting of EM and the need for maintenance therapy remains to be validated in large well-designed studies [21]. A similar conundrum exists for the use of other immunosuppressive agents, such as azathioprine, mycophenolate, and intravenous gammaglobulins, which have been used in conjunction with corticosteroids in the treatment of EM [22-24].

In our two patients, the first patient likely had a subtype of EM known as idiopathic hypereosinophilic syndrome (HES). This syndrome is characterized by absolute eosinophil count greater than 1.5×105/L lasting for more than six months in the absence of any known cause of hypereosinophilia and with evidence of multi-organ involvement directly attributable to the eosinophilia or otherwise unexplained in the clinical setting [4,13,25]. In our second case presentation, the patient did not have a sustained level of peripheral eosinophilia during all of his medical encounters as such it remains unclear whether he had an undifferentiated form EM or HES. Similar case presentations of EM without significant levels of peripheral eosinophils have been documented in few cases across the medical literature [26-29]. The underlying mechanism of HES is postulated to be a primary disorder of myelopoiesis or an overproduction of eosinophilopoietic cytokines by lymphocytes [25]. The most common clinical manifestations include dermatologic, pulmonary, and gastrointestinal features with predominant pruritis, dermatitis, asthma, cough, dyspnea, abdominal pain, vomiting, and diarrhea [30]. However some case series report cardiac involvement in up to 40-50% of patients with HES [31]. Corticosteroid therapy in HES has been successfully documented in published case reports with induced complete or partial responses at 1 month in 85% of patients following monotherapy; most patients remained on maintenance doses with a median of 10 mg prednisone equivalent daily dose for 2 months to 20 years [4,30,32]. It remains uncertain however why our patients failed to respond to the proposed therapy. One possible explanation includes the late initiation of therapy secondary to the elusive diagnosis of EM and by which time myocardial injury maybe irreversible. In addition, sub-therapeutic initiation and or maintenance doses, or treatment resistant fulminant eosinophilic myocarditis may have been key factors in the observed treatment response. In our second case presentation, poor tolerance to corticosteroids and a lack of maintenance therapy were key factor as well.

## Conclusion

EM remains a rare and likely under-diagnosed subtype of myocarditis. The key features of this disease include myocardial injury in the setting of non-contributory coronary artery disease. Endomyocardial biopsy remains the definitive gold standard for diagnosis of noninfectious EM [8]. Non-invasive cardiac imaging in the setting of peripheral eosinophilia can be strongly suggestive of EM with potential for earlier diagnosis. Failure to diagnose EM and the delay of therapy may lead to irreversible myocardial injury. The therapies of EM have yet to be validated in large prospective studies.

## Consent

Written informed consent was obtained from both patients for publication of this case report and any accompanying images. Copies of the written consent are available for review by the Editor-in-Chief of this journal.

## Abbreviations

ANCA: Anti-neutrophil cytoplasmic antibody; CK: Creatine kinase; CRP: C-reactive protein; CT: Computed tomography; ECG: Electrocardiogram; ECP: Eosinophil cationic protein; EF: Ejection fraction; EM: Eosinophilic myocarditis; HES: Hypereosinophilic syndrome; IV: Intravenous; LV: Left ventricle; MRI: Magnetic resonance imaging; NYHA class: New York Heart Association Classification; WBC: White blood cell.

## Competing interests

The authors declare that they have no competing interests.

## References

[B1] Liu P Baughman K Myocarditis L Braunwald’s Heart Disease: A Textbook of Cardiovascular Medicine 2012 9 Philadelphia: Elsevier Saunders 1595 1610

[B2] Kawano S Kato J Kawano N Yoshimura Y Masuyama H Fukunaga T Sato Y Maruyama H Mihara K Ueda A Toyoda K Imamura T Kitamura K Clinical features and outcomes of eosinophilic myocarditis patients treated with prednisolone at a single institution over a 27-year period Intern Med 2011 6 975 981 10.2169/internalmedicine.50.4079 21532219

[B3] Amini R Nielsen C Eosinophilic myocarditis mimicking acute coronary syndrome secondary to idiopathic hypereosinophilic syndrome: a case report J Med Case Reports 2010 6 40 10.1186/1752-1947-4-40 PMC2830978 20181108

[B4] Ali A Straatman L Allard M Ignaszewski A Eosinophilic myocarditis: case series and review of literature Can J Cardiol Vol 2006 6 14 1233 1237 10.1016/S0828-282X(06)70965-5 PMC2569073 17151774

[B5] Bleakley C McEneaney D Eosinophilic myocarditis presenting as acute myocardial infarction J Cardiovasc Med 2010 6 000 000 10.2459/JCM.0b013e32833758e7 20407381

[B6] Talierco CP Olney BA Lie JT Myocarditis related to drug hypersensitivity Mayo Clin Proc 1985 6 463 468 10.1016/S0025-6196(12)60870-2 4010343

[B7] Japanese Circulation Society Task Force Committee on Acute and Chronic Myocarditis Guidelines for diagnosis and treatment of myocarditis (JCS 2009) 2009 http://www.j-circ.or.jp/guideline/pdf/JCS2009_izumi_h.pdf

[B8] Caforio A Pankuweit S Arbustini E Current state of knowledge on aetiology, diagnosis, management, and therapy of myocarditis: a position statement of the European Society of Cardiology Working Group on Myocardial and Pericardial Diseases Eur Heart J 2013 6 33 2636 2648 10.1093/eurheartj/eht210 23824828

[B9] Rezaizadeh H Sanchez-Ross M Kaluski E Klapholz M Haider B Gerula C Acute eosinophilic myocarditis: Diagnosis and treatment Acute Card Care 2010 6 31 36 10.3109/17482940903578998 20201659

[B10] Fragkouli K Mitselou A Boumba V Michalis L Vougiouklakis T An autopsy case of necrotizing eosinophilic myocarditis causing left ventricular wall rupture Am J Forensic Med Pathol 2008 6 4 354 357 10.1097/PAF.0b013e3181859fe3 21516488

[B11] Weller PF Bubley GJ The idiopathic hypereosinophilic syndrome Blood 1994 6 2759 2779 8180373

[B12] Turan AA Karayel F Akyildiz EU Ozdes T Yilmaz E Pakis I Sudden death due to eosinophilic endomyocardial diseases: three case reports Am J Forensic Med Pathol 2008 6 354 357 10.1097/PAF.0b013e3181859fe3 19259026

[B13] Herzog CA Snover DC Staley NA Acute necrotizing eosinophilic myocarditis Br Heart J 1984 6 343 348 10.1136/hrt.52.3.343 6466521 PMC481637

[B14] Burke AP Saenger J Mullick F Virmani R Hypersensitivity myocarditis Arch Pathol Lab Med 1991 6 8 764 769 1863186

[B15] From AM Maleszewski JJ Rihal CS Current status of endomyocardial biopsy Mayo Clin Proc 2011 6 11 1095 1102 10.4065/mcp.2011.0296 22033254 PMC3203000

[B16] Shanes JG Ghali J Billingham ME Ferrans VJ Fenoglio JJ Edwards WD Interobserver variability in the pathologic interpretation of endomyocardial biopsy results Circulation 1987 6 2 401 405 10.1161/01.CIR.75.2.401 3802444

[B17] Arima M Kanoh T Kawano Y Oigawa T Yamagami S Matsuda S Serum levels of eosinophil cationic protein in patients with eosinophilic myocarditis Int J Cardiol 2002 6 1 97 99 10.1016/S0167-5273(02)00074-8 12104073

[B18] Magnani JW Dec GW Myocarditis: current trends in diagnosis and treatment Circulation 2006 6 6 876 890 10.1161/CIRCULATIONAHA.105.584532 16476862

[B19] Friedrich MG Sechtem U Schultz-Menger J Holmwang G Alakija P Cooper LT White JA Abdel-Aty H Gutberlet M Prasad S Aletras A Laissy JP Paterson I Filipchuk N Kumar A Pauschinger M Liu P Cardiovascular magnetic resonance in myocarditisa JACC White paper 2009 6 17 1475 1487 10.1016/j.jacc.2009.02.007 PMC2743893 19389557

[B20] Tani H Amano Y Tachi M Machida T Mizuno K Kumita S T2-weighted and delayed enhancement MRI of eosinophilic myocarditis: relationship with clinical phases and global cardiac function Jpn J Radiol 2012 6 10 824 831 10.1007/s11604-012-0130-3 22956366

[B21] Yanagisawa T Inomata T Watanabe I Maekawa E Mizutani T Shinagawa H Koitabashi T Takeuchi I Izumi T Clinical significance of corticosteroid therapy for eosinophilic myocarditis Int Heart J 2011 6 110 113 10.1536/ihj.52.110 21483171

[B22] Aggarwal A Bergin P Jessup P Kaye D Hypersensitivity myocarditis presenting as cardiogenic shock J Heart Lung Transplant 2001 6 1241 1244 10.1016/S1053-2498(01)00313-8 11704488

[B23] Chau EM Chow WH Chiu CS Wang E Treatment and outcome of biopsy-proven fulminant myocarditis in adults Int J Cardiol 2006 6 3 405 406 10.1016/j.ijcard.2005.07.082 16297469

[B24] Cooper LT Zehr KJ Biventricular assist device placement and immunosuppression as therapy for necrotizing eosinophilic myocarditis Nat clin Pract Cardiovasc Med 2005 6 10 544 548 10.1038/ncpcardio0322 16186853

[B25] Klion AD Noel P Akin C Law MA Gilliland DG Cools J Metcalfe DD Nutman TB Elevated serum tryptase levels identify a subset of patients with a myeloproliferative variant of idiopathic hypereosinophilic syndrome associated with tissue fibrosis, poor prognosis, and imatinib responsiveness Blood 2003 6 12 4660 12676775 10.1182/blood-2003-01-0006

[B26] Blauwet LA Breen JF Edwards WD Klarich KW Atypical presentation of eosinophilic endomyocardial disease Mayo Clin Proc 2005 6 8 1078 1084 10.4065/80.8.1078 16092589

[B27] Fuzellier JF Chapoutot L Torossian PF Metz D Baehrel B Mitral valve replacement in idiopathic eosinophilic endocarditis without peripheral eosinophilia J Card Surg 2005 6 5 472 474 10.1111/j.1540-8191.2005.200460.x 16153283

[B28] Priglinger U Drach J Ullrich R Baumgartner H Huber K Maurer G Idiopathic eosinophilic endomyocarditis in the absence of peripheral eosinophilia Leuk Lymphoma 2002 6 1 215 218 10.1080/10428190210184 11908733

[B29] Sohn IS Park JC Chung JH Kim KH Ahn Y Jeong MH A case of acute eosinophilic myopericarditis presenting with cardiogenic shock and normal peripheral eosinophil count Korean J Intern Med 2006 6 2 136 140 10.3904/kjim.2006.21.2.136 16913446 PMC3890738

[B30] Ogbogu PU Bochner BS Butterfield JH Gleich GJ Huss-Marp J Kahn JE Leiferman KM Nutman TB Pfab F Ring J Rothenberg ME Roufosse F Sajous MH Sheikh J Simon D Simon HU Stein ML Wardlaw A Weller PF Klion AD Hypereosinophilic syndrome: a multicenter, retrospective analysis of clinical characteristics and response to therapy J Allergy Clin Immunol 2009 6 6 1319 25 E3 10.1016/j.jaci.2009.09.022 19910029 PMC2829669

[B31] Ommen SR Seward JB Tajik AJ Clinical and echocardiographic features of hypereosinophilic syndromes Am J Cardiol 2000 6 110 113 10.1016/S0002-9149(00)00841-9 10867107

[B32] Roehrl MH Alexander MP Hammond SB Ruzinova M Wang JY O’Hara CJ Eosinophilic myocarditis in hypereosinophilic syndrome Am J Hematol 2011 6 7 607 608 10.1002/ajh.21943 21681785

